# Higher Risk of Suicide on Milestone Birthdays: Evidence from Japan

**DOI:** 10.1038/s41598-019-53203-4

**Published:** 2019-11-12

**Authors:** Tetsuya Matsubayashi, Myoung-jae Lee, Michiko Ueda

**Affiliations:** 1 Osaka School of International Public Policy, Osaka University, 1-31 Machikaneyama, Toyonaka, Osaka 560-0043 Japan; 2 Department of Economics, Korea University, 145 Anam-ro, Sungbuk-gu, Seoul, 02841 South Korea; 3 Faculty of Political Science and Economics, Waseda University, Building No.3 1-6-1, Nishiwaseda, Shinjuku-ku, Tokyo, 169-8050 Japan

**Keywords:** Risk factors, Epidemiology

## Abstract

Recent studies suggest that the risk of suicide is higher during and around birthdays. The so-called “birthday blues” might be stronger on birthdays at milestone ages (e.g., 20, 30, 40), as these symbolic ages might represent occasions for existential stock-taking that may highlight failures and underachievement in life. Moreover, in some countries (including Japan), certain symbolic birthdays come with the expectation of celebration with family and friends, and thus such special birthdays may elevate the birthday blues if there is nobody to celebrate the occasion with. This study examines the possibility that there are more suicides on milestone birthdays than on other birthdays or days other than birthdays, using approximately one million individual death records between 1974 and 2014 in Japan. Graphical analysis and Poisson regression analysis showed that suicides occurred more frequently on milestone birthdays when people turn 20, 30, 40, and 60. This pattern was predominately observed in men. Our findings suggest that it is crucial for health professionals and family members to pay close attention to vulnerable individuals as their birthdays approach. In particular, individuals are at a higher risk when birthdays coincide with occasions of social significance, including the ages of adulthood (age 20) and retirement (age 60).

## Introduction

Is the risk of suicide higher on one’s birthday? This question is known as the “birthday blues” hypothesis and recently, large population-based data studies strongly support it^1–4^. Daily suicide rates were estimated in Switzerland^1^, Japan^2,3^, England, and Wales^4^ and these studies found that the suicide risk was 6–40% higher on birthdays than other days. Some argue that suicide risk is elevated on birthdays as birthdays can be a source of stress and depression in vulnerable individuals^4,5^. However, despite these findings, the precise etiology of this increase remains unclear^6^.

The influence of birthday blues on suicide may be particularly high when people reach a symbolic or milestone age, such as 20, 30, and 40. Milestone birthdays become occasions to reappraise one’s life^7^. People are more likely to search for existential meaning when approaching the final year of a decade in age (e.g., 19, 29, 39)^8^. Individuals at a milestone age change how they evaluate life, placing more weight on health than daily emotional experiences^9^. The findings of these studies suggest that when people recognize that they have failed in life or anticipate failure in achieving their longstanding goals at milestone ages, they may find little meaning in life and become preoccupied with feelings of hopelessness, which may result in a higher risk of suicide at milestone ages. Indeed, compared to other ages, the frequency of suicide is higher at milestone ages^7^.

Building on the research on birthday blues and milestone ages, this study used large-scale data from Japan to test the hypothesis that suicide is more likely to occur on milestone birthdays than other days and birthdays. Japan is an interesting case to study because there are unique milestone ages in the later stages of life that have been historically celebrated as longevity celebrations. Such special milestone birthdays (i.e. ages 60, 65, 70, 77, 80, 88, 90, 99, and 100) have a particular name and associated meaning. For example, age 70 is called “koki” in Japanese and its name is based on an ancient Chinese poem. These longevity birthdays are typically celebrated with family members and relatives. Given their significance as social events, we would expect that these occasions may heighten the birthday blues or lead to the “broken-promise effect”^10^, if one has nobody to celebrate such special birthdays with.

This is the first study to examine whether the frequency of suicide is higher on birthdays at milestone ages than other days or birthdays. Past studies considered whether there is a peak in suicide at symbolic ages or on birthdays, but no study has investigated whether there is a noticeable jump in suicide counts on the birthday at a milestone age. The current study is closely related to a recent study on suicides and accidents on birthdays in Japan^2^, which used the same dataset as the current study. However, the previous study did not distinguish milestone birthdays from other birthdays and thus did not explore what role the cultural and societal meaning attached to these special birthdays might play in birthday suicides. Our analysis used the national death registry that included all suicide deaths in Japan, thus enabling us to track daily patterns of suicide on birthdays and non-birthdays at any age.

## Methods

This study used the mortality records of the Vital Statistics of Japan between 1974 and 2014 collected by the Ministry of Health, Labour and Welfare. The data was originally collected for administrative purposes and the Ministry made the individual data anonymous and removed any identifying information before making them available for analysis. Individual death records contained in the data were made available only for research purposes and due to the confidentiality agreement with the Ministry, we are not allowed to share the data. Informed consent and an approval from an ethics committee was not necessary because the data were collected by the third party for administrative purposes and the anonymous version was released for this study.

The individual death records are based on the death certificates issued by physicians and cover all reported deaths in Japan. The information in the dataset includes the date of birth, date of death, and underlying cause of death based on the International Classification of Diseases (ICD) 8/9 standard (1974 to 1994) and the ICD-10 standard (1995 to the present). This study analyzed deaths by suicide classified as E950-E959 (ICD-8/9) and X60-X84 (ICD-10).

The analysis excluded the following suicide cases. First, deaths of non-Japanese citizens and individuals whose place of death was outside Japan were excluded from the study. Second, we excluded records that did not include information on the date of birth and/or the date of death because our analysis required both. Third, death records that did not contain valid information on the place where the death was reported were also removed because this indicates that some of the circumstances surrounding the deaths were unknown, and thus the accuracy of the records may be compromised. Fourth, our analysis did not include the deaths of individuals aged less than 15 and more than 90 years because the number of suicide deaths in these age categories were particularly small. Finally, we excluded the death records of individuals whose date of birth or the date of death is on February 29 in order to simplify our analysis.

For each death record, we calculated age in days at death using the date of birth and death, taking into account leap years. For example, age in days at death equals 7305 when a person died on their 20^th^ birthday. This variable ranged from 5478 (15^th^ birthday) to 32872 (90^th^ birthday) days at death. For each value of age in days at death over the 41-year period, we aggregated the frequency of suicides for all records as well as aggregated suicides by sex. Thus, the unit of analysis in our graphical as well as statistical analysis described below was age in days.

We examined whether more suicides occurred on milestone birthdays than other birthdays and non-birthdays in two ways. Both analyses were based on the data of all suicide counts as well as sex-specific suicide counts. First, we conducted graphical analysis and plotted the frequencies of suicides against the age in days. The birthdays at each milestone age in days from 20 to 90 were marked to determine whether any increase exists on milestone birthdays. Second, using a Poisson regression model, we regressed the frequency of suicides at each age in days on 76 dummy variables. These dummy variables were coded as 1 if the age in days at death coincided with one’s birthday (between 15 and 90 years), and zero otherwise. Thus, the baseline category was all non-birthdays. This model specification allowed us to estimate whether people were more likely to die on their birthday, and whether this birthday effect was stronger on milestone than non-milestone birthdays. In addition to the dummy variables of birthdays, we included in the Poisson regression model, the scale of age in days and its squared term, and dummy variables for each chronological decade such as 20 s, 30 s, 40 s, 50 s, 60 s, 70 s, and 80 s (including 90 years old) by setting 10 s as the baseline category. The scale of age in days and its squared term were expected to capture a potential curvilinear relationship with the frequency of suicides, while the dummies of chronological decades were expected to control for the potential influences of life stages on suicide. To facilitate the interpretation of the Poisson regression results, the coefficients were converted to Incidence Rate Ratios (IRR) by exponentiating the Poisson regression coefficients associated with the 76 dummy variables of birthdays. IRRs measure the rate at which suicide occurs on birthdays relative to that occurring on non-birthdays. IRRs greater than 1.0 mean that suicide is more likely to occur on birthdays than non-birthdays, while those smaller than 1.0 mean the opposite. We used Stata 15 by StataCorp for the analyses.

## Results

Our data contained 1,000,631 suicides that occurred between 1974 and 2014, of which 676,147 were by male individuals and 330,184 by female individuals. The average number of total suicides aggregated by the age in days at death was 32.4 (SD = 17.6), and the maximum number was 95. The maximum was observed at 18,993 days in age after birth, which was the 52^nd^ birthday. The average number of male suicides was 21.8 (SD = 13.7), and the maximum number was 73. The average number of female suicides was 10.6 (SD = 5.6), and the maximum number was 34.

**Figure 1:**
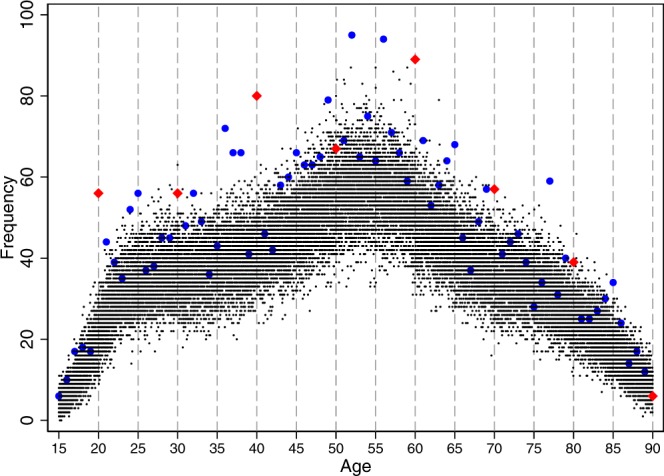
The frequency of suicides by age in days at death. Notes: the frequency of suicides by the age in days at death shown. The red diamonds and blue circles denote the frequency of suicides on milestone birthdays at ages ending in 0 and non-milestone birthdays, respectively. All other black dots denote the frequency of suicides on non-birthdays. Source: the Vital Statistics of Japan, 1974–2014.

Figure 1 plots the frequency of suicides by the age in days. The horizontal axis denotes the scale of age in days from 5478 (15^th^ birthday) to 32872 (90^th^ birthday) days. For readability, we displayed the birthdays between ages 15 and 90 in five-year intervals using labels of age in years. For example, we used 15 instead of 5478 to mark the 15^th^ birthday, which occur 5478 days after birth. The vertical dashed lines denote the birthdays between ages 20 and 90 in five-year intervals. The red diamonds denote the frequency of suicides on milestone birthdays at ages from 20 and 30 to 90 and the blue circles denote the frequency of suicides on non-milestone birthdays. All other black dots denote the frequency on non-birthdays. Figure 1 shows visible jumps on the milestone birthday ages of 20, 40, and 60; additional noticeable jumps were observed on the milestone birthdays at ages 30 and 50. Other noticeable jumps occur on the birthday at ages of 36, 52, 56, 63, 65, and 77.

**Figure 2:**
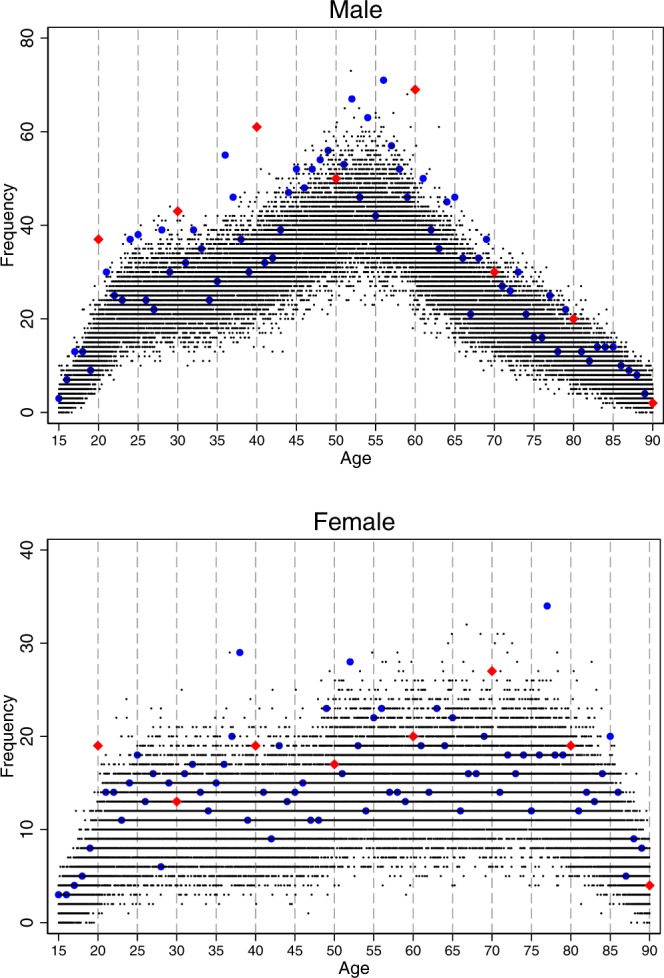
The frequency of suicides by age in days at death and sex. Notes: the frequency of suicides by males and females is shown at the top and the bottom, respectively. The red diamonds and blue circles denote the frequency of suicides on milestone birthdays at ages ending in 0 and non-milestone birthdays, respectively. All other black dots denote the frequency of suicides on non-birthdays. Source: the Vital Statistics of Japan, 1974–2014.

Figure 2 presents the suicide frequency for male and female individuals separately. The jump at the milestone birthday at age 20 was observed for both sexes. Aside from this similarity, patterns of suicide were distinct between male and female individuals. Male suicide frequencies were high at the milestone birthdays at ages 30, 40, and 60 and other birthdays at ages 36, 52, 56, 59, and 63. In contrast, there was no observable increase in female suicide on these birthdays; instead, the frequency of female suicide was high on the birthdays at ages 38 and 77. Female suicides also exhibited some increases on the birthdays at ages 37 and 68. The female suicide frequency was highest at age 77, one of the special milestone birthdays in Japan.

**Figure 3:**
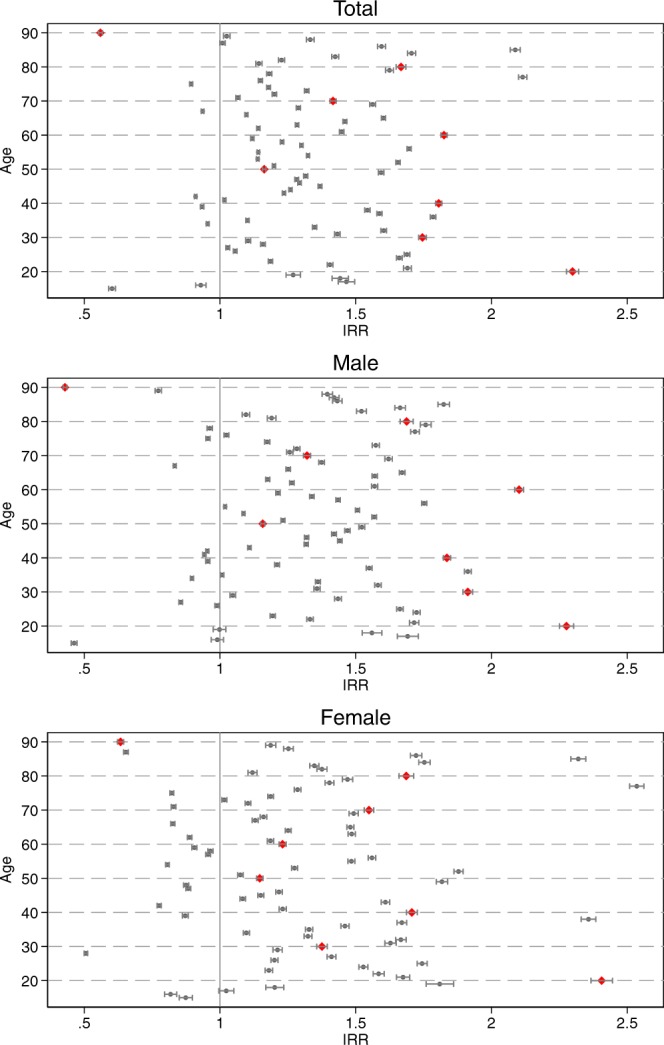
Incidence Rate Ratios for suicides for each birthday by sex. Note: The Incidence Rate Ratios (IRRs) are based on the estimation results of Poisson regression models shown. The models include 76 dummy variables for each birthday between the ages 15–90, the age in days and its squared term, and dummy variables for each chronological age-decade from the 20 s to 80 s, using the 10 s as a baseline category. The y-axis indicates the birthday dummy variables; the baseline category was all non-birthdays. The x-axis shows the IRR with the 95% confidence interval. The red diamonds denote milestone birthdays that end in 0. Source: the Vital Statistics of Japan, 1974–2014.

Figure 3 reports the estimation results of Poisson regression models using the total and sex-specific suicides. The models include 76 dummy variables for each birthday between ages 15 and 90, the age in days and its squared term, and dummy variables for each chronological age-decade from the 20 s to 80 s, using the 10 s as a baseline category. For readability, Fig. 3 displays IRRs for only 76 dummies converted from the estimated coefficients of the Poisson regression model. Full estimation results are available online as Supplementary Tables S1 and S2. The y-axis indicates each of the birthday dummy variables with all non-birthdays as the baseline category. The IRRs denote the rate at which suicide occurs on milestone and other birthdays relative to the rate at which suicides occurs on non-birthdays. The x-axis shows the sizes of IRRs with the 95% confidence interval. The red diamonds denote milestone birthdays at ages 20, 30, and 40 to 90.

The top panel shows that most birthdays had IRRs greater than one, indicating that suicides are more likely to occur on birthdays than other days. Figure 3 also indicates that the risk of suicide is higher on some birthdays than others, but not all of them are milestone birthdays. Among milestone birthdays, the most conspicuous increase was observed at the age of 20. The IRR estimated using data for both sexes at age 20 was 2.30, whereas the average IRR for all the other birthdays was 1.32. For male individuals, the milestone ages 30, 40, and 60 also showed a high risk of suicide. However, such increases were not observed among female individuals. Female suicides exhibit a particularly high risk of suicide at age 77 with an IRR of 2.54.

## Discussion

This study analyzed whether individuals are more likely to die by suicide on milestone birthdays than non-milestone birthdays or other days. Milestone birthdays can represent an occasion for existential stock-taking^7–9^, which can heighten feelings of failure and perceptions of underachievement, particularly in vulnerable individuals. In addition, milestone birthdays, especially those in old age, are often social and festive events that are expected to be celebrated with families and friends. The social significance of milestone birthdays may heighten “birthday blues”^5^ or initiate the “broken promises” effect^10^ for individuals that have nobody with whom to celebrate their birthday.

Using 41 years of population data on approximately 1 million suicide deaths in Japan between 1974 and 2014, we found that there were far more male suicides on the milestone birthdays of ages 20, 30, 40, and 60. This pattern differed in female suicides, which tended to concentrate on birthdays of ages 20 and 77. These tendencies were observed even after controlling for age and life-stage effects.

Our study contributes to the literature by documenting the existence of milestone birthday suicides using large-scale national data. While many previous studies have shown that the risk of suicide is higher on one’s birthday, no previous study has explicitly investigated which birthday poses the highest risk, because the previous studies examined the prevalence of birthday suicides only by age groups^2–4,11^. The result of the present study clearly indicates that not just any birthday, but a very few special birthdays, are associated with an elevated risk for individuals, especially among male individuals.

The observed high frequencies of suicide on the 20^th^ birthday may be explained by the fact that it represents the age of adulthood. In Japan, on becoming 20 years old, individuals are conferred with various rights and responsibilities, including drinking and smoking. The 20^th^ birthday is typically celebrated with families and friends, as well as in a ceremony on a national holiday (“coming-of-age day”) in mid-January. Thus, turning 20 years old is a socially recognized significant milestone in the Japanese society. It is possible that some young individuals feel that they have already lived long enough or reluctant to start a new chapter of their life beyond their 20^th^ birthday, especially if life had been tough up to that point. However, this observation remains speculative, as we do not have direct evidence on the motives behind these deaths.

As for the large increase in male suicides on their 60th birthdays, this could be related to the retirement age in Japan or the longevity celebration for the 60th birthdays. As of 2005, 95% of companies with more than 1,000 employees set the retirement age at 60^12^, with some Japanese companies setting the retirement day as one day before the employee’s birthday. Although some workers keep working after their retirement, they typically work as short-term contractors with a significantly reduced pay^13^. Thus, the 60th birthday constitutes a major life event for many male workers in Japan where men tend to work outside whereas women tend to stay at home with or without a part-time job. It also marks the beginning of the traditional 60-year cycle in the East Asian tradition, which is called “kanreki” in Japanese. It is the first of the traditional longevity birthdays and is the one that is most highly recognized by the public. Such a festive and social occasion might elicit despair in some individuals. However, the fact that an increase in suicides at age 60 is observed only among men suggests that the retirement age could be a more relevant factor than the longevity birthday.

It is harder to interpret the observed increases on other birthdays, including the 30th and 40th birthdays for men and the 77th birthday for women. The 77th birthday is one of the traditionally cerebrated longevity birthdays, but it is not as significant an event as the 60th or 70th birthdays. As for the 30th and 40th birthdays, some individuals may regard them as milestones, as they mark the end of their 20 s and 30 s. However, there is no particularly important cultural or social meaning attached to these birthdays in Japan. In addition, the observed increases on other non-milestone birthdays are even harder to explain, and thus more research is necessary in order to understand why individuals choose to mark their birthday with their death.

The following limitations were observed in this study. First, our analysis was based on data from Japan, and thus, our findings may not be applicable in other countries, as different cultures may view and treat birthdays differently^14,15^. However, the tradition of longevity celebration exists in other East Asian countries. For example, the 60th birthday is celebrated as “Hwangap” in Korea and “Jiazi” in China. Future studies should investigate whether the elderly population is more likely to engage in suicidal behavior in the aftermath of socially recognized events and celebrations, especially in a culture where family ties have been historically and traditionally strong but are presently eroding. Second, we only examined suicides on one’s actual birthday; however, it is possible that risks for suicide are elevated on the days surrounding one’s birthday^3,4,11^, and such possibilities were not explicitly tested in this study. Similarly, due to the nature of our data, we only examined the date of death, not the date on which the decedents attempted to kill themselves, which can lead to an under- or over-estimation of our estimates. For example, if an individual attempted suicide on the evening of their birthday, and died the following day, their death would not be recorded as a birthday suicide, resulting in an under-estimation. In contrast, if an individual attempted suicide on the eve of their birthday but died on their birthday, it would result in an over-estimation. Third, we do not have data on why these individuals killed themselves on their birthday, and we could not explain the reasons behind the observed patterns in this study. More qualitative studies are necessary in order to fully understand why some people choose to die on their birthday.

Our study has important policy implications. It is crucial for health professionals and family members to pay close attention to vulnerable individuals as their birthdays approach. In particular, our findings suggest that individuals are at a higher risk when birthdays coincide with occasions of social significance, including the ages of adulthood (age 20) and retirement (age 60). Given that health professionals should have their patients’ dates of birth as records, it might be helpful to approach high-risk individuals prior to their birthday. Employing a method that does not require such patients to make a hospital visit, such as sending post cards and text messages, may prove beneficial^16,17^. The effectiveness of these methods to prevent birthday suicides should be evaluated in future research.

## Supplementary information

Supplementary Tables

## Data Availability

The data that we used in this study are individual death records and its use is restricted to researchers who obtain written approval from the Ministry of Health, Labour, and Welfare of Japan. Under the conditions of our contract with the Ministry, we are not allowed to share the data with third parties. Information on how to obtain these restricted data can be found at: https://www.mhlw.go.jp/english/database/link.html.
