# Supplementary material for: Higher Risk of Suicide on Milestone Birthdays: Evidence from Japan
Source: Sci Rep. 2019 Nov 12;9:16642. doi: 10.1038/s41598-019-53203-4 (PMC6851132; doi:10.1038/s41598-019-53203-4)
Supplement: Supplementary file 1 — Supplementary Tables [file 41598_2019_53203_MOESM1_ESM.pdf]

Higher Risk of Suicide on Milestone Birthdays: Evidence from Japan

Tetsuya Matsubayashi[1]

Myoung-jae Lee[2]

Michiko Ueda[3*]

[1] Osaka School of International Public Policy, Osaka University, 1-31 Machikaneyama, Toyonaka, Osaka 560-0043, Japan <matsubayashi@osipp.osaka-u.ac.jp>

[2] Department of Economics, Korea University, 145 Anam-ro, Sungbuk-gu, Seoul 02841, South Korea <myoungjae@korea.ac.kr>

[3] Faculty of Political Science and Economics, Waseda University, Building No.3 1-6-1 Nishiwaseda, Shinjuku-ku, Tokyo 169-8050, Japan <mueda@waseda.jp>

*Corresponding author: Michiko Ueda, Faculty of Political Science and Economics, Waseda University; Building No. 3, 1-6-1 Nishiwaseda, Shinjuku-ku, Tokyo 169-8050; tel: +81-3-3208-1176; mueda@waseda.jp

Supplementary Tables

Supplementary Table S1: Estimated coefficients of the Poisson regression analysis

	(1)<br>Total	(2)<br>Male	(3)<br>Female
Birthdays			
15	-0.507***	-0.771***	-0.135***
	(0.011)	(0.012)	(0.014)
16	-0.073***	-0.010	-0.201***
	(0.011)	(0.012)	(0.014)
17	0.382***	0.526***	0.023*
	(0.011)	(0.012)	(0.014)
18	0.367***	0.445***	0.184***
	(0.011)	(0.012)	(0.014)
19	0.239***	-0.002	0.593***
	(0.011)	(0.012)	(0.014)
20.	0.832***	0.823***	0.878***
	(0.005)	(0.006)	(0.008)
21	0.525***	0.540***	0.516***
	(0.004)	(0.005)	(0.007)
22	0.341***	0.287***	0.460***
	(0.004)	(0.005)	(0.007)
23	0.171***	0.178***	0.166***
	(0.003)	(0.004)	(0.006)
24	0.507***	0.545***	0.424***
	(0.003)	(0.004)	(0.006)
25	0.524***	0.509***	0.557***
	(0.003)	(0.004)	(0.005)
26	0.055***	-0.011***	0.183***
	(0.003)	(0.004)	(0.006)
27	0.029***	-0.156***	0.344***
	(0.003)	(0.004)	(0.006)
28	0.147***	0.361***	-0.681***
	(0.004)	(0.004)	(0.006)
29	0.099***	0.046***	0.192***
	(0.004)	(0.005)	(0.007)
30	0.557***	0.649***	0.319***
	(0.004)	(0.005)	(0.007)
31	0.359***	0.306***	0.487***
	(0.004)	(0.004)	(0.007)
32	0.472***	0.459***	0.511***
	(0.003)	(0.004)	(0.006)
33	0.299***	0.308***	0.281***
	(0.003)	(0.004)	(0.005)
34	-0.046***	-0.109***	0.093***
	(0.003)	(0.003)	(0.005)
35	0.097***	0.008**	0.284***
	(0.003)	(0.003)	(0.005)
36	0.579***	0.649***	0.379***
	(0.003)	(0.003)	(0.005)
37	0.462***	0.438***	0.513***
	(0.003)	(0.004)	(0.005)
38	0.434***	0.191***	0.858***
	(0.003)	(0.004)	(0.006)
39	-0.068***	-0.046***	-0.137***

	(0.004)	(0.004)	(0.006)
40	0.591***	0.608***	0.535***
	(0.003)	(0.004)	(0.006)
41	0.016***	-0.059***	0.208***
	(0.003)	(0.003)	(0.005)
42	-0.094***	-0.047***	-0.253***
	(0.003)	(0.003)	(0.005)
43	0.212***	0.103***	0.476***
	(0.003)	(0.003)	(0.005)
44	0.231***	0.276***	0.080***
	(0.003)	(0.003)	(0.005)
45	0.314***	0.366***	0.140***
	(0.003)	(0.003)	(0.005)
46	0.257***	0.277***	0.197***
	(0.003)	(0.003)	(0.005)
47	0.249***	0.351***	-0.124***
	(0.003)	(0.003)	(0.005)
48	0.274***	0.385***	-0.133***
	(0.003)	(0.003)	(0.005)
49	0.466***	0.420***	0.598***
	(0.003)	(0.004)	(0.006)
50	0.151***	0.146***	0.137***
	(0.003)	(0.003)	(0.005)
51	0.181***	0.209***	0.073***
	(0.003)	(0.003)	(0.005)
52	0.504***	0.450***	0.631***
	(0.002)	(0.003)	(0.005)
53	0.130***	0.083***	0.243***
	(0.002)	(0.003)	(0.004)
54	0.281***	0.410***	-0.215***
	(0.002)	(0.003)	(0.004)
55	0.132***	0.019***	0.395***
	(0.002)	(0.003)	(0.004)
56	0.529***	0.561***	0.445***
	(0.002)	(0.003)	(0.004)
57	0.262***	0.361***	-0.045***
	(0.002)	(0.003)	(0.005)
58	0.206***	0.292***	-0.036***
	(0.003)	(0.003)	(0.005)
59	0.113***	0.194***	-0.099***
	(0.003)	(0.003)	(0.005)
60	0.602***	0.743***	0.208***
	(0.003)	(0.004)	(0.005)
61	0.370***	0.451***	0.171***
	(0.003)	(0.004)	(0.005)
62	0.132***	0.235***	-0.119***
	(0.003)	(0.003)	(0.005)
63	0.250***	0.162***	0.396***
	(0.003)	(0.003)	(0.005)
64	0.379***	0.451***	0.224***
	(0.003)	(0.003)	(0.004)
65	0.471***	0.513***	0.392***
	(0.003)	(0.003)	(0.005)
66	0.093***	0.224***	-0.191***
	(0.003)	(0.003)	(0.005)
67	-0.066***	-0.182***	0.122***
	(0.003)	(0.004)	(0.005)

68	0.254***	0.318***	0.149***
	(0.003)	(0.004)	(0.005)
69	0.446***	0.483***	0.400***
	(0.003)	(0.004)	(0.006)
70	0.348***	0.278***	0.437***
	(0.004)	(0.005)	(0.006)
71	0.064***	0.229***	-0.187***
	(0.004)	(0.005)	(0.005)
72	0.183***	0.249***	0.098***
	(0.003)	(0.004)	(0.005)
73	0.277***	0.454***	0.016***
	(0.003)	(0.004)	(0.005)
74	0.165***	0.161***	0.171***
	(0.003)	(0.004)	(0.004)
75	-0.112***	-0.045***	-0.195***
	(0.003)	(0.004)	(0.005)
76	0.139***	0.024***	0.251***
	(0.003)	(0.004)	(0.005)
77	0.749***	0.541***	0.930***
	(0.004)	(0.005)	(0.005)
78	0.167***	-0.039***	0.339***
	(0.004)	(0.005)	(0.006)
79	0.485***	0.564***	0.385***
	(0.005)	(0.006)	(0.007)
80	0.511***	0.523***	0.523***
	(0.006)	(0.007)	(0.008)
81	0.134***	0.174***	0.113***
	(0.005)	(0.007)	(0.007)
82	0.204***	0.091***	0.319***
	(0.005)	(0.006)	(0.007)
83	0.354***	0.420***	0.298***
	(0.005)	(0.006)	(0.006)
84	0.534***	0.509***	0.561***
	(0.004)	(0.006)	(0.006)
85	0.736***	0.601***	0.842***
	(0.004)	(0.006)	(0.006)
86	0.467***	0.359***	0.544***
	(0.005)	(0.006)	(0.006)
87	0.009*	0.351***	-0.425***
	(0.005)	(0.007)	(0.007)
88	0.287***	0.333***	0.225***
	(0.005)	(0.007)	(0.007)
89	0.025***	-0.258***	0.171***
	(0.006)	(0.008)	(0.008)
90	-0.580***	-0.846***	-0.456***
	(0.007)	(0.009)	(0.009)
Age in days	0.112***	0.126***	0.094***
	(0.001)	(0.001)	(0.002)
Age in days squared	-0.001***	-0.001***	-0.001***
	(0.000)	(0.000)	(0.000)
Dummy for 20-29	0.530***	0.513***	0.521***
	(0.013)	(0.015)	(0.019)
Dummy for 30-39	0.244***	0.221***	0.211***
	(0.017)	(0.019)	(0.027)
Dummy for 40-49	0.230***	0.253***	0.063*
	(0.020)	(0.023)	(0.033)
Dummy for 50-59	0.379***	0.415***	0.216***

	(0.023)	(0.026)	(0.038)
Dummy for 60-69	0.322***	0.299***	0.352***
	(0.025)	(0.028)	(0.042)
Dummy for 70-79	0.464***	0.348***	0.645***
	(0.026)	(0.030)	(0.044)
Dummy for 80-89	0.478***	0.372***	0.610***
	(0.028)	(0.033)	(0.048)
Constant	0.870***	0.266***	0.030
	(0.021)	(0.024)	(0.032)
N	27759	27759	27759

Note: The estimation results of Poisson regression models where the outcome is total suicides aggregated by the age in days at death are shown. The numbers in the table refer to birthdays and the baseline category was all non-birthdays.

Source: the Vital Statistics of Japan, 1974-2014.

Supplementary Table S2. IRR converted from the estimated coefficients in Supplementary Table S1.

	(1)<br>Total	(2)<br>Male	(3)<br>Female
Birthdays			
15	0.602***	0.463***	0.874***
	(0.006)	(0.005)	(0.012)
16	0.929***	0.990	0.818***
	(0.010)	(0.012)	(0.011)
17	1.466***	1.691***	1.023*
	(0.015)	(0.020)	(0.014)
18	1.443***	1.560***	1.202***
	(0.015)	(0.018)	(0.017)
19	1.270***	0.998	1.809***
	(0.014)	(0.012)	(0.026)
20.	2.299***	2.276***	2.406***
	(0.011)	(0.013)	(0.020)
21	1.691***	1.715***	1.674***
	(0.007)	(0.009)	(0.012)
22	1.406***	1.332***	1.584***
	(0.005)	(0.006)	(0.010)
23	1.186***	1.194***	1.180***
	(0.004)	(0.005)	(0.007)
24	1.660***	1.724***	1.528***
	(0.005)	(0.007)	(0.008)
25	1.689***	1.663***	1.745***
	(0.005)	(0.006)	(0.009)
26	1.056***	0.989***	1.201***
	(0.003)	(0.004)	(0.007)
27	1.029***	0.855***	1.411***
	(0.004)	(0.003)	(0.008)
28	1.158***	1.435***	0.506***
	(0.004)	(0.006)	(0.003)
29	1.104***	1.047***	1.212***
	(0.005)	(0.005)	(0.009)
30	1.746***	1.913***	1.376***
	(0.007)	(0.009)	(0.010)
31	1.432***	1.358***	1.628***
	(0.005)	(0.006)	(0.011)
32	1.603***	1.582***	1.666***
	(0.005)	(0.006)	(0.010)
33	1.349***	1.361***	1.324***
	(0.004)	(0.005)	(0.007)
34	0.955***	0.897***	1.097***
	(0.003)	(0.003)	(0.006)
35	1.101***	1.008**	1.328***
	(0.003)	(0.003)	(0.007)
36	1.785***	1.913***	1.460***
	(0.005)	(0.006)	(0.008)
37	1.587***	1.550***	1.670***
	(0.005)	(0.005)	(0.009)
38	1.543***	1.210***	2.358***
	(0.005)	(0.005)	(0.014)
39	0.934***	0.955***	0.872***
	(0.003)	(0.004)	(0.006)
40	1.806***	1.836***	1.707***
	(0.006)	(0.007)	(0.010)

41	1.016***	0.943***	1.231***
	(0.003)	(0.003)	(0.007)
42	0.910***	0.954***	0.776***
	(0.002)	(0.003)	(0.004)
43	1.236***	1.109***	1.610***
	(0.003)	(0.003)	(0.008)
44	1.260***	1.318***	1.084***
	(0.003)	(0.004)	(0.005)
45	1.369***	1.441***	1.151***
	(0.003)	(0.004)	(0.006)
46	1.293***	1.319***	1.218***
	(0.003)	(0.004)	(0.006)
47	1.283***	1.420***	0.883***
	(0.004)	(0.005)	(0.005)
48	1.316***	1.470***	0.876***
	(0.004)	(0.005)	(0.005)
49	1.594***	1.523***	1.818***
	(0.005)	(0.006)	(0.011)
50	1.163***	1.158***	1.147***
	(0.003)	(0.004)	(0.006)
51	1.199***	1.232***	1.075***
	(0.003)	(0.004)	(0.005)
52	1.656***	1.568***	1.879***
	(0.004)	(0.004)	(0.009)
53	1.139***	1.087***	1.275***
	(0.003)	(0.003)	(0.006)
54	1.324***	1.506***	0.807***
	(0.003)	(0.004)	(0.004)
55	1.141***	1.019***	1.484***
	(0.003)	(0.003)	(0.006)
56	1.697***	1.752***	1.560***
	(0.004)	(0.005)	(0.007)
57	1.300***	1.435***	0.956***
	(0.003)	(0.004)	(0.004)
58	1.229***	1.338***	0.965***
	(0.003)	(0.004)	(0.005)
59	1.119***	1.214***	0.906***
	(0.003)	(0.004)	(0.005)
60	1.825***	2.102***	1.231***
	(0.006)	(0.008)	(0.007)
61	1.448***	1.570***	1.186***
	(0.004)	(0.006)	(0.006)
62	1.141***	1.265***	0.888***
	(0.003)	(0.004)	(0.004)
63	1.284***	1.176***	1.485***
	(0.003)	(0.004)	(0.007)
64	1.460***	1.570***	1.251***
	(0.004)	(0.005)	(0.006)
65	1.602***	1.671***	1.480***
	(0.004)	(0.005)	(0.007)
66	1.097***	1.251***	0.826***
	(0.003)	(0.004)	(0.004)
67	0.936***	0.833***	1.130***
	(0.003)	(0.003)	(0.006)
68	1.289***	1.374***	1.160***
	(0.004)	(0.005)	(0.006)
69	1.562***	1.621***	1.492***

	(0.005)	(0.007)	(0.009)
70	1.416***	1.321***	1.549***
	(0.006)	(0.007)	(0.009)
71	1.066***	1.257***	0.829***
	(0.004)	(0.006)	(0.004)
72	1.201***	1.283***	1.103***
	(0.004)	(0.006)	(0.005)
73	1.320***	1.574***	1.016***
	(0.004)	(0.007)	(0.005)
74	1.179***	1.174***	1.187***
	(0.004)	(0.005)	(0.005)
75	0.894***	0.956***	0.823***
	(0.003)	(0.004)	(0.004)
76	1.149***	1.024***	1.286***
	(0.004)	(0.005)	(0.006)
77	2.115***	1.718***	2.535***
	(0.008)	(0.008)	(0.013)
78	1.182***	0.962***	1.403***
	(0.005)	(0.005)	(0.008)
79	1.625***	1.758***	1.470***
	(0.007)	(0.010)	(0.010)
80	1.667***	1.688***	1.687***
	(0.009)	(0.012)	(0.014)
81	1.144***	1.190***	1.120***
	(0.006)	(0.008)	(0.008)
82	1.227***	1.096***	1.376***
	(0.006)	(0.007)	(0.009)
83	1.425***	1.521***	1.348***
	(0.006)	(0.009)	(0.009)
84	1.706***	1.664***	1.753***
	(0.008)	(0.010)	(0.011)
85	2.088***	1.824***	2.320***
	(0.009)	(0.011)	(0.014)
86	1.595***	1.432***	1.722***
	(0.007)	(0.009)	(0.011)
87	1.009*	1.421***	0.654***
	(0.005)	(0.009)	(0.004)
88	1.333***	1.395***	1.252***
	(0.007)	(0.010)	(0.009)
89	1.025***	0.773***	1.187***
	(0.006)	(0.006)	(0.010)
90	0.560***	0.429***	0.634***
	(0.004)	(0.004)	(0.006)
Age in days	1.119***	1.135***	1.098***
	(0.001)	(0.002)	(0.002)
Age in days squared	0.999***	0.999***	0.999***
	(0.000)	(0.000)	(0.000)
Dummy for 20-29	1.699***	1.671***	1.684***
	(0.022)	(0.024)	(0.031)
Dummy for 30-39	1.277***	1.247***	1.234***
	(0.022)	(0.024)	(0.033)
Dummy for 40-49	1.259***	1.288***	1.065*
	(0.026)	(0.030)	(0.035)
Dummy for 50-59	1.461***	1.515***	1.241***
	(0.034)	(0.040)	(0.047)
Dummy for 60-69	1.380***	1.349***	1.422***
	(0.034)	(0.038)	(0.059)

Dummy for 70-79	1.590***	1.416***	1.907***
	(0.042)	(0.043)	(0.085)
Dummy for 80-89	1.613***	1.451***	1.841***
	(0.046)	(0.048)	(0.088)
N	27759	27759	27759

Note: The table contains the estimated coefficients reported in Supplementary Table S1 that were converted to the Incidence Rate Ratios (IRRs).
Source: the Vital Statistics of Japan, 1974-2014.
